# Local response function estimation in spherical deconvolution for comprehensive tissue representation using diffusion MRI

**DOI:** 10.1162/IMAG.a.95

**Published:** 2025-08-01

**Authors:** Siebe Leysen, Ahmed Radwan, Frederik Maes, Stefan Sunaert, Daan Christiaens

**Affiliations:** Department of Electrical Engineering, ESAT/PSI, KU Leuven, Leuven, Belgium; Medical Imaging Research Center, University Hospitals Leuven, Leuven, Belgium; Department of Imaging and Pathology, Translational MRI, KU Leuven, Leuven, Belgium; Department of Radiology, University Hospitals Leuven, Leuven, Belgium

**Keywords:** diffusion MRI, spherical deconvolution, tractography, glioma

## Abstract

Diffusion MRI (dMRI) plays a crucial role in studying tissue microstructure and fibre orientation. Due to the intricate nature of the dMRI signal, end users require representations that provide a straightforward interpretation. Currently, these representations rely on tissue-average estimations or simplified tissue models and are hence limited in their applicability to pathology. In this study, we propose a novel approach called LoRE-SD—a local response function estimation in spherical deconvolution. LoRE-SD minimises assumptions about tissue microstructure to improve the reconstruction of dMRI data in the presence of pathology. This is achieved by introducing a general signal representation that spans the most common multi-compartment microstructure models used in neuroimaging. Leveraging spherical deconvolution, LoRE-SD provides accurate estimations of the local fibre orientations, allowing tractography in the healthy and pathological brain. We evaluate this approach using simulations and in vivo data from a healthy volunteer and from patients with glioma. Comparing the results quantitatively with the state-of-the-art, we find that LoRE-SD accurately reconstructs fibre orientations across the brain while also significantly improving glioma reconstruction and fibre bundle estimation. Additionally, the tissue representation in LoRE-SD facilitates the generation of various image contrasts, including response function anisotropy and contrasts accentuating intra-axonal, extra-axonal, and free water spaces, which enables a more flexible approach for tractography. In conclusion, LoRE-SD introduces a framework for estimating a data-driven, local representation of tissue microstructure with minimal prior assumptions. This approach provides a new way to represent the human brain, pathology, and other organs using dMRI and opens avenues for defining novel image contrasts, which may benefit tractography.

## 1 Introduction

Diffusion-weighted magnetic resonance imaging (dMRI) is a medical imaging modality that enables the non-invasive exploration of brain tissue microstructure and connectivity (Le Bihan et al., 1989). dMRI probes the random motion of water molecules, known as Brownian motion, which is hindered or restricted by cell membranes (Le Bihan, 2003). By measuring the diffusion of water in biological tissue, dMRI offers unique insights into the microstructural characteristics and structural connectivity of brain white matter (WM) on a subvoxel scale (Alexander et al., 2019). It has become an invaluable tool for researching neurodegenerative conditions (Acosta-Cabronero & Nestor, 2014; Mito et al., 2018), brain tumours (Charles-Edwards & Souza, 2006; Gihr et al., 2021), and multiple sclerosis (Inglese & Bester, 2010).

dMRI captures the orientation-dependent diffusion of water molecules at various diffusion strengths (b-values) uniformly across the sphere, achieving high angular resolution (HARDI). The raw diffusion signal is complex and challenging to analyse, necessitating the use of mathematical models and representations to interpret the data and to understand the diffusion characteristics of water molecules in tissue. While the preferred model or representation in dMRI analysis depends on the radiologist’s needs and diagnostic objectives, a shared assumption in most models that aim to characterise tissue microstructure or fibre orientations is the adoption of the spherical convolution model (Anderson, 2005; Jespersen et al., 2007; Luca et al., 2020; Tournier et al., 2004):

S(b,g)=(ℋ*P)(b,g).

The signal S(b,g) with b-value b and gradient direction g is approximated as the spherical convolution of a voxel-wise, axially symmetric response function ℋ(b,g⋅n) and fibre orientation distribution function (ODF) P(n), where n is the fibre orientation. In the orthonormal basis of real, symmetric Spherical Harmonics $Y_{\ell }^{m}\left(\text{n}\right)$ of order $\ell =0,\;2,...,\ell_{max}$ and index m=−ℓ,...,ℓ , this simplifies to an outer product per SH order ℓ:

$$\forall \ell \;:\;S_{\ell }=h_{\ell }p_{\ell }^{⊤},$$

where $h_{\ell }^{⊤}=\left[\cdots h_{\ell ,b}\cdots \right]$ (indexed in *b*) and $p_{\ell }^{⊤}=\left[\cdots p_{\ell }^{m}\cdots \right]$ (indexed in *m*) (Christiaens et al., 2020).

Within the spherical convolution paradigm, there exists a broad array of approaches, depending on the constraints and functional forms they employ for the response function and the ODF (Christiaens et al., 2020). Biophysical models for the response function are common to quantitatively assess tissue microstructure (Jespersen et al., 2007; Kaden, Kelm, et al., 2016; Kaden, Kruggel, et al., 2016; Novikov, Veraart, et al., 2018; Reisert et al., 2017). Such models take a functional form parameterised by a limited set of biophysical parameters, for example a two-compartment model of the intra-axonal and extra-axonal space or a three-compartment model that also includes a free water compartment. These compartments are required to model the non-monoexponential diffusion signal arising from the intra- and extra-axonal spaces (Assaf & Cohen, 1998; Buckley et al., 1999; Jespersen et al., 2007; Lampinen et al., 2020; Novikov et al., 2019). They are modelled using axial and radial diffusivities and are assumed to have Gaussian diffusion processes (assuming no compartmental exchange).

These biophysical models are highly non-linear in nature, making it difficult to provide a stable (unconstrained) model fit using only linear diffusion tensor encoding (Coelho et al., 2019, 2022; Jelescu et al., 2016; Lampinen et al., 2020; Novikov et al., 2019). Within such data, the degeneracy in parameter estimation is alleviated by introducing constraints (Kaden, Kruggel, et al., 2016; H. Zhang et al., 2012). In addition, the AMICO framework (Daducci et al., 2015) alleviates the burdensome fitting of biophysical models by decoupling the ODF from the microstructure and reformulating the problem as a linear system. Dictionary-based approaches have also shown to accurately estimate microstructure (Rensonnet et al., 2019) and ODFs (Baete et al., 2019). ODF fingerprinting (Baete et al., 2019) constructs a dictionary of ODFs (generated using simulated dMRI signals) to find the ODF in the dictionary that best matches the measured ODF. As such, ODF accuracy is improved in the presence of noise and smaller crossing angles can be resolved. However, they are all rooted in a biophysical model of diffusion, which is tailored to white matter in the adult brain. In the presence of severe pathology, such as glioma, white matter models may fail to accurately represent the tissue and may cause model parameters to lose their biophysical significance.

An alternative approach is to employ a data-driven response function representation rather than a biophysical model. Constrained Spherical Deconvolution (CSD) (Tournier et al., 2004, 2007) and its multi-tissue extension (MSMT-CSD) (Jeurissen et al., 2014) directly estimate the SH representation of the response function from the dMRI data. The lack of a clear biophysical meaning in data-driven methods is subordinate to accurate ODF estimation, which is crucial for tractography. CSD and MSMT-CSD use canonical response functions to this end. The tissue response is assumed to be independent of the fibre bundle, such that a brain-wide white matter (and grey matter (GM) and cerebrospinal fluid (CSF) in MSMT-CSD) response function is used and the spherical convolution operation has only one unknown, being the ODF (Christiaens et al., 2017; Jeurissen et al., 2014; Tournier et al., 2013). Here too, it can be argued that in the presence of (severe) pathology, canonical response functions fail to accurately fit to the diffusion signal. In addition, the microstructural organisation within glioma is very heterogeneous, making it infeasible to design (robust heuristics for) glioma-average response functions.

In this case, a local, data-driven representation of the response function (Anderson, 2005; Daducci et al., 2015; Schultz & Groeschel, 2013) is argued to be beneficial for modelling tissue variability (Jespersen et al., 2007; Schilling et al., 2019) and, additionally, may enhance tractography (Christiaens et al., 2015; Daducci et al., 2016; Reisert et al., 2017; Veraart et al., 2018). One of the earliest examples of such approach, FORECAST, parameterises the response function in every voxel using a single perpendicular diffusivity $\lambda_{⊥}$ (Anderson, 2005), and it has inspired other techniques that improve the angular resolution of crossing fibres by introducing ODF sparsity (Schultz & Groeschel, 2013). The representation of the response function in these methods is often parameterised with very few degrees of freedom, providing an elegant and simple representation tailored to the healthy brain. However, accurately modelling heterogeneous tissue, such as glioma, requires more complex representations, and the required number of compartments cannot be defined a priori (Novikov, Kiselev, et al., 2018). Furthermore, while these representations are highly specific, they are sensitive to the estimation of these few parameters (Novikov, Kiselev, et al., 2018; Schultz & Groeschel, 2013), similar to the biophysical models.

In this work, we introduce a novel, local representation of the response function within the spherical deconvolution framework. We represent the response function as a linear combination of axially symmetric Gaussian basis functions, which provides a robust and flexible representation for capturing complex microstructural characteristics of brain tissue. During fitting, we jointly optimise the local response function representation and the ODF (Section 2). Using simulations and in vivo glioma data (Section 3), our approach is rigorously evaluated against the state-of-the-art (Section 4). Finally, we demonstrate the possibilities of our method in contrast generation and its impact on tractography (Section 4.4) and discuss our findings, highlighting implications and considerations (Section 5).

## 2 Methods

### 2.1 LoRE-SD: Local response function estimation in spherical deconvolution

Building on the idea of a local microstructure representation, we fit a local response function and ODF to dMRI data using the spherical convolution operation. As validated in vivo (Christiaens et al., 2020), the signal in each voxel S is accurately represented as a single spherical convolution of a voxel-level response function $H=\left[h_{0}\cdots h_{\ell_{max}}\right]$ and ODF $p^{⊤}=\left[p_{0}\;p_{2}^{⊤}\cdots p_{\ell_{max}}^{⊤}\right]$. To accommodate an a priori undefined number of tissue compartments, the response function is represented as a linear mixture model of compartments, assuming no inter-compartmental exchange. Each compartment is defined as an axially symmetric Gaussian basis function G, parameterised by the b-value, the elevation angle α, and the axial and radial diffusivity, denoted as $\lambda_{∥}$ and $\lambda_{⊥}$, respectively (Anderson, 2005):

$$G\left(b,\alpha \text{|}\lambda_{∥},\lambda_{⊥}\right)=e^{-b\lambda_{⊥}}e^{-b\left(\lambda_{∥}-\lambda_{⊥}\right){\text{cos}}^{2}\alpha }.$$ (1)

The basis function G is described as a matrix of zonal harmonics coefficients G that has the same dimensions as the response function matrix H. These coefficients are calculated using a Legendre polynomial fit with respect to cos  α∈[−1, 1] (Anderson, 2005).

**Fig. 1.**
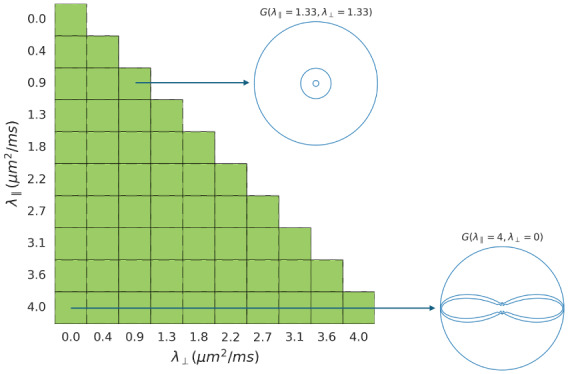
A grid of axial and radial diffusivities is used to parameterise the set of Gaussian basis functions of the response function. During optimisation, a weight is assigned to each basis function and the weighted mixture model then represents the local response function.

To represent the response function, we define a set of basis functions on a discrete grid of axial and radial diffusivities, each linearly spaced in the range $\left[0,\;4\right]\;\mu m^{2}\text{​}/\text{​}ms$ (Fig. 1). During optimisation, a weight $f_{\lambda_{∥},\lambda_{⊥}}\in \left[0,\;1\right]$ is associated with every basis function $G_{\lambda_{∥},\lambda_{⊥}}$. The response function, therefore, represents a linear mixture model of microstructural compartments, where the number of compartments is not fixed a priori. Its mathematical formulation is shown in Eq. (5) and Eq. (6). The objective is to jointly estimate, in each voxel, the mixture fractions $f_{\lambda_{∥},\lambda_{⊥}}$ that represent the local response function and the SH coefficients that represent the ODF.

As the spherical deconvolution is ill-conditioned, a non-negativity constraint on the ODF (Eq. 4) is imposed to stabilise the fit and reduce spurious peaks (Tournier et al., 2007). To further reduce spurious ODF peaks, the objective function includes a regularisation term to promote more isotropic response functions (Eq. 2), defined as the squared $l_{2}$ -norm of all response function coefficients with SH order ℓ>0 . More isotropic response functions indirectly promote ODF sparsity, which is a commonly used regularisation term (Daducci et al., 2014; Landman et al., 2012; Michailovich et al., 2011; Schultz & Groeschel, 2013). ODF sparsity requires evaluation of the ODF in a dense set of directions, which leads to increased computational complexity compared with the regularisation introduced here. Finally, we enforce unit normalisation of the ODF to alleviate the undefined isotropic scaling factor that arises when the response function and ODF are estimated simultaneously (Eq. 3).

The resulting optimisation problem is formulated as

$$\underset{H,p}{\text{min}}\sum_{\ell \in \left\{0,2,...,\ell_{max}\right\}}{\left\|S_{\ell }-N_{\ell }H_{\ell }{\text{p}}_{\ell }^{⊤}\right\|}_{F}^{2}+\lambda \sum_{\ell >0}{\left\|H_{\ell }\right\|}_{2}^{2},$$ (2)

subject to

$${\text{p}}_{0}=\frac{1}{\sqrt{4\pi }}\text{,}$$ (3)

Qp≥0, (4)

$$H=S_{0}\sum_{\lambda_{∥}=0}^{\lambda_{∥}^{\left(max\right)}}\sum_{\lambda_{⊥}=0}^{\lambda_{∥}}f_{\lambda_{∥},\lambda_{⊥}}G\left(\lambda_{∥},\lambda_{⊥}\right)\text{ with }0\leq f_{\lambda_{∥},\lambda_{⊥}}\leq 1$$ (5)

$$\;\sum_{\lambda_{∥}=0}^{\lambda_{∥}^{\left(max\right)}}\sum_{\lambda_{⊥}=0}^{\lambda_{∥}}f_{\lambda_{∥},\lambda_{⊥}}\text{​}=1.$$ (6)

In this formulation, $N_{l}$ is a normalisation factor $\left(N_{l}=\sqrt{\frac{4\pi }{2\ell +1}}\right)$ and Q evaluates SH amplitudes in 300 directions uniformly distributed along the sphere. In line with the literature, we employ a maximum SH order of $\ell_{max}=8$ (Tournier et al., 2013). The regularisation parameter λ has been fixed to $\lambda =10^{-3}$ in all experiments. We have validated that this provides an adequate trade-off between ODF accuracy and root mean square error (RMSE) using simulations and in vivo brain data, see Appendix A.1. In addition, the grid size for the response function representation has been fixed to a 10 by 10 square grid of uniformly distributed diffusivities between $\left[0,\;4\right]\;\;\mu m^{2}\text{​}/\text{​}ms$ . The size of this grid was also validated using simulations and in vivo data (cf. Appendix A.2).

The Gaussian mixture model, which defines the response function, is initially set up uniformly and used to perform a single CSD iteration. This iteration optimises the ODF based on the response function, resulting in an (ODF, response function)-pair that serves as the initialisation for LoRE-SD. This proves to provide a simple, yet stable initialisation procedure, regardless of the underlying tissue microstructure.

### 2.2 Image contrasts

The representation of a localised response function as a Gaussian mixture model allows the derivation of contrast maps. To highlight specific features or microstructures in a dataset, weights are allocated to each Gaussian basis function. These weights accentuate the ($\lambda_{∥},\lambda_{⊥}$)-pairs of interest, resulting in a contrast-specific weight matrix W (contrast matrix). The contrast is constructed as the inner product of W and the Gaussian mixture model in every voxel $f_{\lambda_{∥},\lambda_{⊥}}$: $\langle W,f_{\lambda_{∥},\lambda_{⊥}}\rangle$. This work introduces four contrast matrices.

The intra-axonal compartment is characterised by negligible radial diffusion. To emphasise intra-axonal space, a contrast matrix $W_{i}$ is constructed with substantial weights assigned at $\lambda_{⊥}=0\;\mu m^{2}\;/\;ms$ , which diminish exponentially as they approach $\lambda_{⊥}=\lambda_{∥}$. This exponential reduction is used to maintain smoothness in the resulting contrast. To highlight free-moving water, it is assumed that free water has axial and radial diffusivities of $\lambda_{∥},\lambda_{⊥}\approx 3\;\mu m^{2}\text{​}/\text{​}ms$ , which is in line with physical theory and experiments (Mills, 1973). To account for minor discrepancies attributable to noise, the weights $W_{fw}$ for free water are set to 1 if the diffusivity is greater than $2.6\;\mu m^{2}\text{​}/\text{​}ms$ . Lastly, the extra-axonal compartment $W_{e}$ ensures that the sum of weights for all basis functions is equal to 1:

$$W_{i}\left(\lambda_{⊥}\right)=e^{-d\lambda_{⊥}}$$ (7)

$$W_{fw}\left(\lambda_{∥},\lambda_{⊥}\right)=\{\begin{array}{cc} 1 & \text{if}\;\lambda_{∥},\lambda_{⊥}\geq 2.6\;\mu m^{2}\text{​}/\text{​}ms\\ 0 & \text{otherwise} \end{array}$$ (8)

$$W_{e}=1-W_{i}-W_{fw},$$ (9)

where the parameter d in the definition of the matrix $W_{i}\left(\lambda_{⊥}\right)$ is used to control the exponential decay for $\lambda_{⊥}>0$ . Setting $d=10\;ms\text{​}/\text{​}\mu m^{2}$ ensures a smooth transition between diffusivities, while exponentially reducing the effect of components with $\lambda_{⊥}>0\;\mu m^{2}\text{​}/\text{​}ms$ .

The fourth contrast sets the weight of each basis function to its fractional anisotropy, which is defined using the eigenvalues $\lambda_{1},\lambda_{2},$ and $\lambda_{3}$ of the diffusion tensor (Basser, 1995):

$$\begin{array}{ccc} FA\left(\lambda_{1},\lambda_{2},\lambda_{3}\right) & =\sqrt{\frac{1}{2}\frac{{\left(\lambda_{1}-\lambda_{2}\right)}^{2}+{\left(\lambda_{2}-\lambda_{3}\right)}^{2}+{\left(\lambda_{3}-\lambda_{1}\right)}^{2}}{\lambda_{1}^{2}+\lambda_{2}^{2}+\lambda_{3}^{2}}}. & \end{array}$$ (10)

This work assumes that diffusion perpendicular to the fibre axis is uniform, such that $\lambda_{1}=\lambda_{∥}$ and $\lambda_{2}=\lambda_{3}=\lambda_{⊥}$. The equation used to determine the weights for the response function anisotropy ($W_{aniso}$ ) simplifies to

$$\begin{array}{ccc} FA\left(\lambda_{∥},\lambda_{⊥}\right)=W_{aniso}\left(\lambda_{∥},\lambda_{⊥}\right) & =\sqrt{\frac{{\left(\lambda_{∥}-\lambda_{⊥}\right)}^{2}}{\lambda_{∥}^{2}+2\lambda_{⊥}^{2}}}. & \end{array}$$ (11)

### 2.3 Tractography

LoRE-SD uses unit-normalised ODFs which renders ODF amplitude inappropriate as a stopping criterion for tractography (Jeurissen et al., 2011; Tournier et al., 2012). Taking this into account, any contrast accentuating white matter (e.g., the intra-axonal compartment fraction or response function anisotropy) can be used as a stopping criterion. Modulating the ODF with these contrasts results in reduced ODF amplitude in isotropic (GM, CSF) regions, enabling early termination of streamline tracking and is similar to the FA stopping criterion used in DTI tractography (Mori & Zijl, 2002). Similar local representations of microstructure have shown to improve tractography (Girard et al., 2017; Reisert et al., 2014), but ODF modulation is limited to the parameters of the biophysical model. The local response function representation introduced by LoRE-SD enables a flexible framework for tractography, where ODF magnitudes can be controlled using custom contrast definitions.

The iFOD2 algorithm (Tournier et al., 2010) is used in combination with inclusion and exclusion volumes of interest (VOI) for bundle-specific tracking, which are automatically segmented (Radwan et al., 2022). To maximise anatomical correctness, anatomy constraints are included using a five tissue type segmentation (R. E. Smith et al., 2012). Constraints do not apply within the manually segmented glioma. The default stopping criterion based on ODF lobe amplitude of 0.05 was used. The inclusion volumes of interest served as the seeding regions.

### 2.4 Optimisation aspects

The optimisation procedure is implemented in Python using the SciPy toolbox (Virtanen et al., 2020). Sequential Least Squares Programming (SLSQP) is opted for, which is a gradient-based iterative solver and can handle both non-linear constraints and bounds. SLSQP is particularly suitable for LoRE-SD due to its ability to manage complex optimisation problems. A relative function tolerance of $10^{-4}$ has proven to be sufficient. Efficiency is improved by providing the analytically derived Jacobian of the objective function. In addition, a suitable response function and ODF are initialised using a single CSD iteration using a uniform distribution of the Gaussian basis functions. Furthermore, since LoRE-SD processes each voxel independently, the optimisation procedure is highly parallellisable, making optimal use of the available hardware. On human brain data (see below) and using 50 threads, LoRE-SD takes 6 minutes and 3 seconds, though further run time optimisations can be made in the future.

## 3 Data and Experiments

The performance of the proposed method is evaluated using the angular correlation of ODF estimations and the RMSE of signal reconstruction. The Angular Correlation Coefficient (ACC) (Anderson, 2005) is used to quantify the similarity between the ODF estimations. For two real, axially symmetric spherical harmonics U and V, the ACC equals

$$ACC\left(U,V\right)=\frac{\sum_{\ell =2}^{\ell_{max}}\sum_{m=-\ell }^{\ell }u_{\ell ,m}v_{\ell ,m}}{{\left[\sum_{\ell =2}^{\ell_{max}}\sum_{m=-\ell }^{\ell }{\left|u_{\ell ,m}\right|}^{2}\right]}^{1/2}{\left[\sum_{\ell =2}^{\ell_{max}}\sum_{m=-\ell }^{\ell }{\left|v_{\ell ,m}\right|}^{2}\right]}^{1/2}}.$$ (12)

RMSE is tracked in every voxel to ensure an accurate modelling of the underlying diffusion signal irrespective of tissue.

### 3.1 Simulations

To assess the accuracy of ODF estimation, single-fibre ($0^{\circ }$) and crossing fibre ($60^{\circ }$) configurations were simulated using apodised Dirac delta functions. The simulated response functions comprise stick, zeppelin, and free water compartments. A default response function using prototypical values for white matter and an alternative response function with increased $D_{e}^{⊥}$ and increased free water fraction are defined as shown in Table 1. Rician noise levels are incorporated at SNRs of 50, 20, and 10. All experiments were compared with MSMT-CSD (Jeurissen et al., 2014), and every configuration was repeated 500 times at each noise level. MSMT-CSD was executed using the ground truth response function as the WM response function.

**Table 1.** Response function definitions used in the simulations.

Parameter	Response function	Alternative response function
$f_{stick}$	0.45	0.3
$f_{zeppelin}$	0.45	0.4
$f_{water}$	0.1	0.3
$D_{a}^{∥}$	$2.2\;\mu m^{2}\text{​}/\text{​}ms$	$2.2\;\mu m^{2}\text{​}/\text{​}ms$
$D_{e}^{∥}$	$2.0\;\mu m^{2}\text{​}/\text{​}ms$	$2.0\;\mu m^{2}\text{​}/\text{​}ms$
$D_{e}^{⊥}$	$0.7\;\mu m^{2}\text{​}/\text{​}ms$	$1.4\;\mu m^{2}\text{​}/\;ms$

A full brain dataset is simulated based on in vivo data to validate tissue modelling using a local response function against tissue-average response functions. Data from one healthy volunteer using a dMRI encoding with $b=0,\;1000,\;2000\;s/mm^{2}$ and corresponding 5, 128, 128 gradient encoding directions were used. Ground truth response functions are estimated from the in vivo data (Dhollander et al., 2019); ground truth ODFs are generated using MSMT-CSD. A forward spherical convolution then generates the noise-free diffusion signal. In line with the above simulation, Rician noise is introduced at SNRs 50, 20, and 10, relative to the mean b=0 signal in WM.

### 3.2 Human brain data

Data were collected following written informed consent and approval by the Ethics Committee Research UZ/KU Leuven (S61759).

#### 3.2.1 Healthy volunteer

A healthy volunteer dataset was acquired on a 3T Philips Achieva MRI with a 32-channel dStream head coil at 2 mm isotropic resolution. Diffusion weightings of b= 0, 1000, and 2000 $s\text{​}/\text{​}mm^{2}$ were applied in 5, 128, and 128 uniformly distributed gradient directions, respectively. TR was set to 4500 ms and TE was set to 85 ms. In addition, a T1w scan with spatial resolution of $0.67\times 0.67\times 0.67\;mm^{3}$ was acquired for tissue segmentation into white matter, cortical grey matter, deep grey matter, and CSF.

#### 3.2.2 Glioma patients

Five low-grade and five high-grade glioma patients were scanned on a 3T Philips Achieva MRI with a 32-channel dStream head coil at 2 mm isotropic resolution. Seven datasets apply diffusion weightings of b= 0, 1200, and 2500 $s\text{​}/\text{​}mm^{2}$ in 5, 128, and 125 directions, respectively. Three datasets apply diffusion weightings of b= 0, 1200, and 2500 $s\text{​}/\text{​}mm^{2}$ in 10, 127, and 123 directions, respectively. TR was set to 4500 ms and TE was set to 85 ms. In addition, a T1w scan was acquired with spatial resolution of $0.9\times 0.9\times 0.9\;mm^{3}$. These data are a random subset of the data used by Radwan et al. (2024). The gliomas were manually segmented by an expert radiologist (A.R.).

### 3.3 Data preprocessing

The healthy volunteer data and glioma data were processed identically. The brain is extracted from the mean $b=0\;s\text{​}/\text{​}mm^{2}$ volume using the Brain Extraction Tool (BET) (S. M. Smith, 2002) from the FMRIB Software Library (FSL). Diffusion-weighted images were denoised (Veraart et al., 2016), corrected for Gibbs ringing (Kellner et al., 2016), eddy currents, and motion and distortion artefacts (Andersson & Sotiropoulos, 2016; Andersson et al., 2003), and finally corrected for bias fields (Y. Zhang et al., 2001).

White matter, grey matter, and CSF response functions were estimated heuristically (Dhollander et al., 2019) excluding any glioma tissue, as there are no heuristics for average glioma response function estimation. These response functions were used to generate ODFs using MSMT-CSD (Jeurissen et al., 2014).

## 4 Results

### 4.1 Simulations

**Fig. 2.**
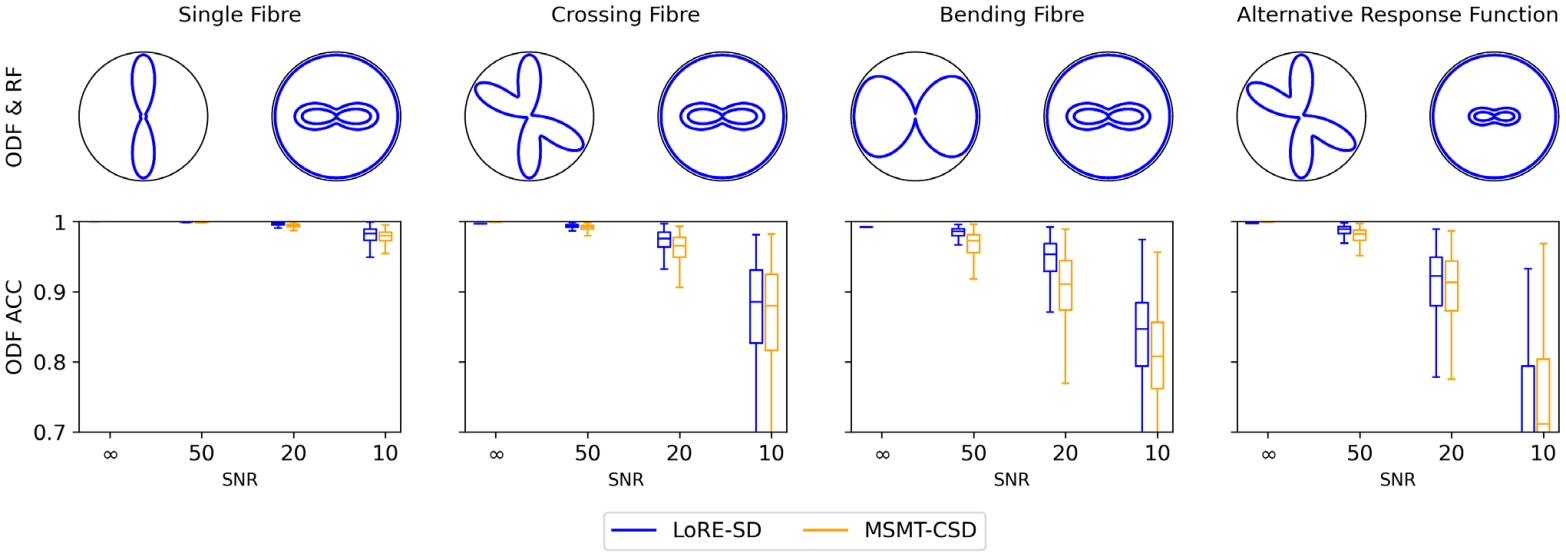
Boxplots of the ACC between the simulated ODF and the ODF estimated with LoRE-SD and MSMT-CSD for different fibre configurations and SNR. Each boxplot comprises 500 noise realisations. LoRE-SD outperforms MSMT-CSD in all configurations except for the alternative response function at SNR 10.

Figure 2 shows the ACC for the ODFs estimated using LoRE-SD and MSMT-CSD. These were assessed for single- and crossing-fibre scenarios under various Rician noise levels. The data indicate a consistent enhancement in ODF accuracy as the signal-to-noise ratio (SNR) increases. Furthermore, the ODF estimations for a single-fibre configuration demonstrate superior accuracy compared with those involving crossing fibres. For SNR values of 20 and above, the average ACC for both LoRE-SD and MSMT-CSD exceeds 0.90 in each configuration, and LoRE-SD outperforms MSMT-CSD. At SNR 10, ODF accuracy drops significantly and MSMT-CSD outperforms LoRE-SD in the alternative response function configuration.

**Fig. 3.**
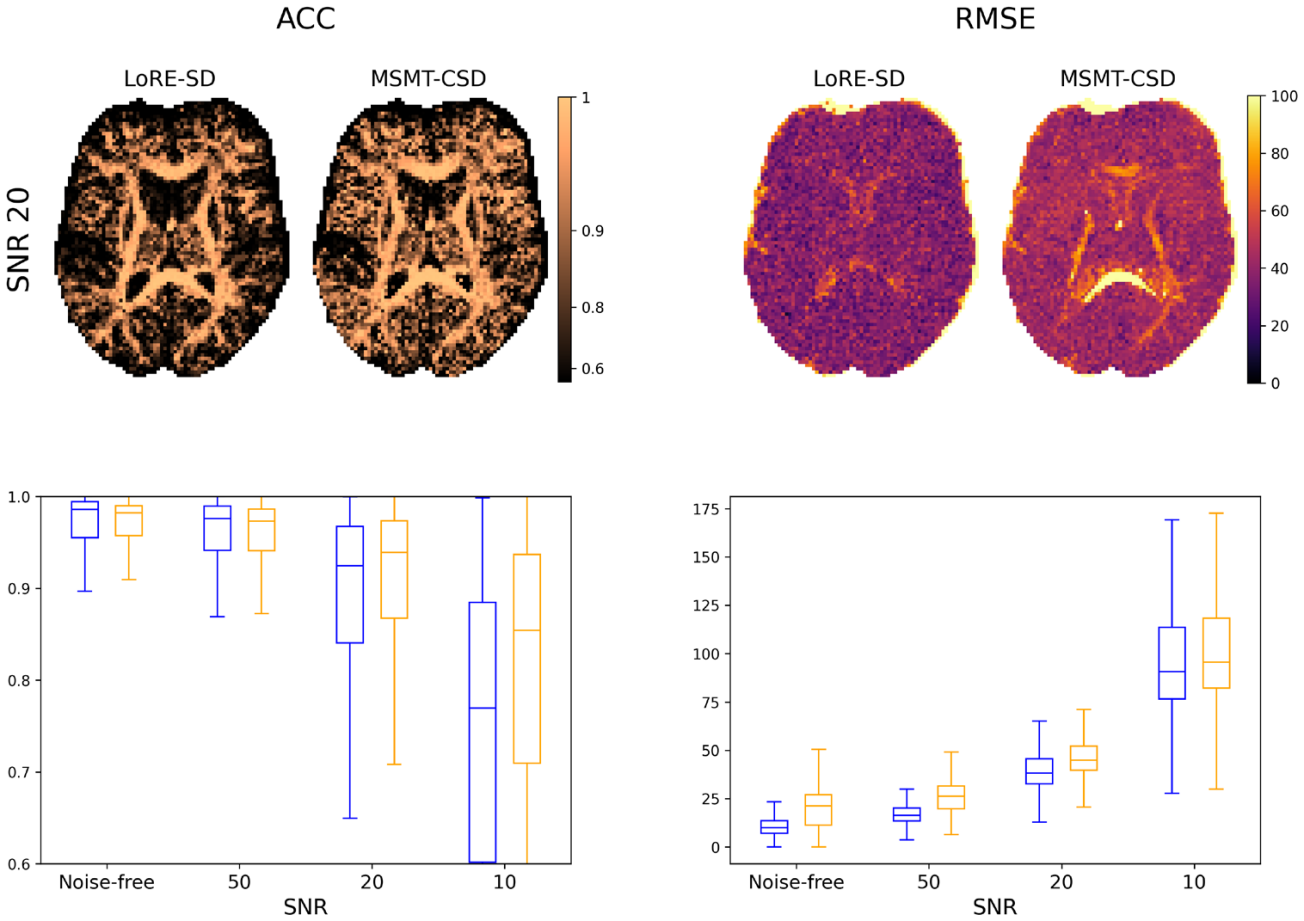
Comparison of LoRE-SD and MSMT-CSD on a simulated axial brain slice showing ACC and RMSE (SNR 20) as well as boxplots summarising full-brain statistics. LoRE-SD and MSMT-CSD provide similar ACC values in WM. At SNR 20 and below, MSMT-CSD outperforms LoRE-SD. Lower RMSE (with respect to the ground truth) and less anatomical structure by LoRE-SD (visualised at SNR 20) indicate better signal modelling in all tissue types, independent of SNR.

Figure 3 shows ACC and RMSE of a single slice at SNR 20. As also evidenced by the boxplots, ACC is comparable with MSMT-CSD in white matter. LoRE-SD is more affected by noise than MSMT-CSD, but ACC remains above 0.9 at SNR 20. The RMSE (with respect to the ground truth) slice displays the clear advantage of voxelwise response functions, as MSMT-CSD exhibits high RMSE within the deep white matter. The advantage of LoRE-SD gradually decreases for decreasing SNR.

### 4.2 In vivo experiments

**Fig. 4.**
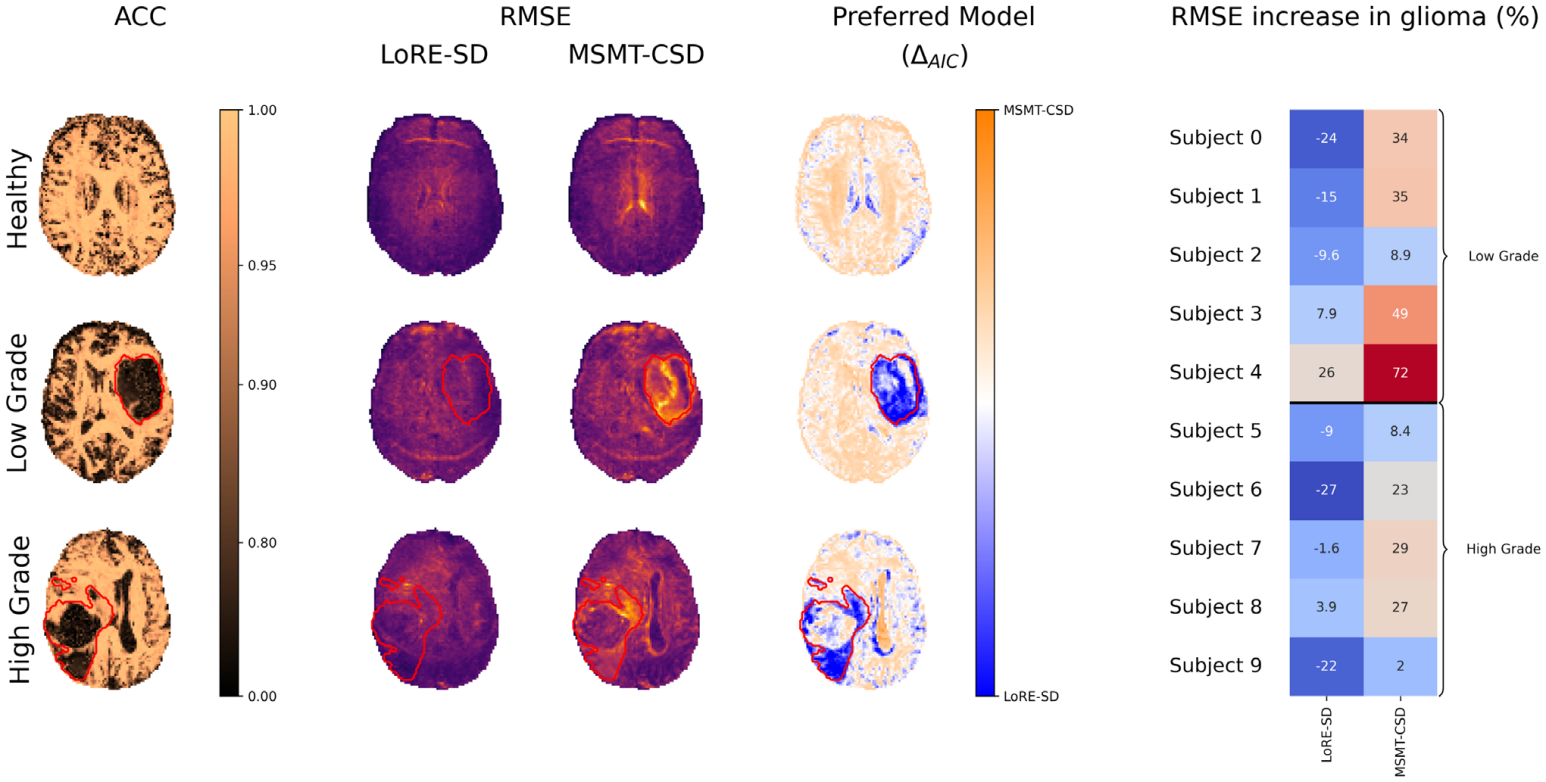
ODF similarity between LoRE-SD and MSMT-CSD estimations assessed by ACC shows close alignment in WM. Residual maps by LoRE-SD are consistently lower than MSMT-CSD, with differences most notably within the glioma region (delineated in red). AIC is used to validate the extra degrees of freedom introduced in LoRE-SD. The preferred model map, which is the difference in AIC values $\Delta_{AIC}=AIC_{LoRE-SD}-AIC_{MSMT-CSD}$ , indicates that LoRE-SD is the preferred model specifically in the glioma regions. Finally, the last column displays the increase in RMSE of glioma tissue with respect to healthy tissue for five low-grade and five high-grade glioma, demonstrating that LoRE-SD consistently models glioma more accurately than MSMT-CSD. Fat shift artefacts are present in the healthy volunteer dataset (anterior) and the low-grade glioma dataset (posterior).

The findings of the simulated data are substantiated by in vivo experiments. Figure 4 illustrates the ACC between the ODF estimations given by LoRE-SD and MSMT-CSD, and the RMSE obtained with each method for three datasets (healthy brain, low-grade glioma, and high-grade glioma). Within WM, the ODF estimations generated by LoRE-SD and MSMT-CSD exhibit high similarity, with the average ACC value exceeding 0.95 across all 11 datasets. LoRE-SD results in reduced RMSE compared with MSMT-CSD, particularly within the glioma region. Moreover, the RMSE obtained with LoRE-SD remains uniform across the entire dataset. Figure 4 also presents the difference in Akaike Information Criterion (AIC) of both methods ($\Delta_{AIC}=AIC_{LoRE-SD}-AIC_{MSMT-CSD}$ ), which is a model selection criterion that offers a relative measure of model quality given the trade-off between goodness-of-fit and model simplicity (Akaike, 1998). LoRE-SD has 45+55−1−1=98 model parameters, representing, respectively, 45 ODF coefficients, 55 Gaussian mixture fractions, 1 constraint on the fractions (sum to unity), and 1 constraint on the ODF (normalisation). MSMT-CSD has 45+1+1=47 model parameters, with 45 coefficients for the WM ODF, 1 for the GM fraction, and 1 for the CSF fraction. LoRE-SD has lower AIC values in the CSF and glioma regions supporting the hypothesis that a local, data-driven response function is superior to canonical response function when modelling heterogeneous tissue such as glioma. Within healthy tissue, RMSE maps of LoRE-SD and MSMT-CSD are similar in magnitude while MSMT-CSD has fewer model parameters, making it the method of choice according to AIC. Lastly, the right column of Figure 4 quantitatively compares the increase in RMSE in glioma tissue compared with healthy tissue for LoRE-SD and MSMT-CSD. Using LoRE-SD, the RMSE values in glioma are on average 2.94% and 11.14% lower than the RMSE in healthy tissue for low- and high-grade glioma, respectively. In MSMT-CSD, RMSE increased by 39.78% and 17.88% in low- and high-grade glioma, respectively.

### 4.3 Contrast generation

**Fig. 5.**
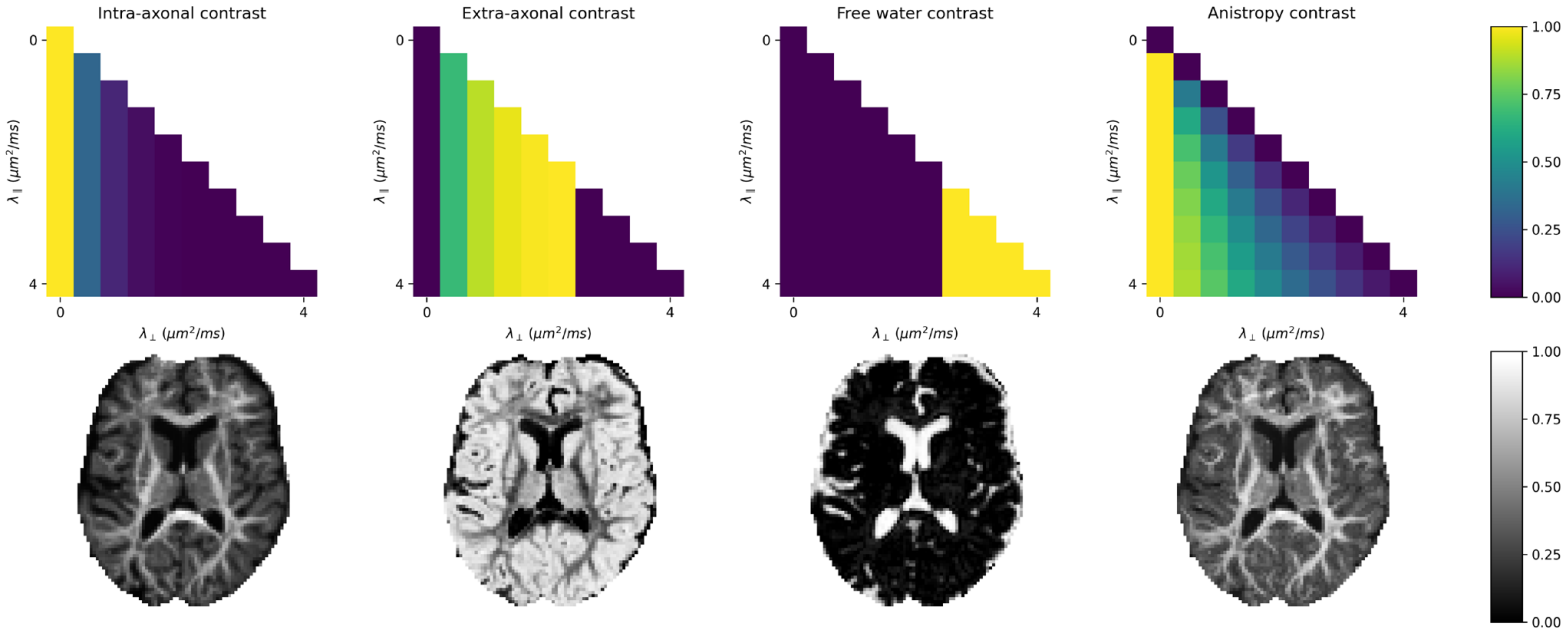
The representation of the response function can be used to generate contrast maps by defining contrast matrices specific to the intra-axonal, extra-axonal, and free water volume fractions. The rightmost column uses fractional anisotropy (determined by $\lambda_{∥}$ and $\lambda_{⊥}$) as a weight for each basis function, which results in a response function anisotropy metric. By calculating the inner product of these matrices (top) with the local response function representations, we obtain the volume fractions (bottom).

As explained in Section 2.2, the response function representation can be used to generate scalar contrast maps by calculating the inner product with a carefully designed contrast matrix. Drawing inspiration from microstructure modelling techniques, we generate contrasts to highlight the intra-axonal, extra-axonal, and free water spaces. In addition, an anisotropy matrix is designed by calculating the FA for each Gaussian basis function. Figure 5 illustrates the predefined weight matrices and resulting contrasts for the intra-axonal, extra-axonal, free water, and anisotropy maps. As hypothesised, the intra-axonal and anisotropy maps exhibit increased intensity in the white matter, particularly in regions of single fibre bundles. The extra-axonal compartment predominantly highlights the parenchyma. The free water compartment accentuates CSF.

**Fig. 6.**
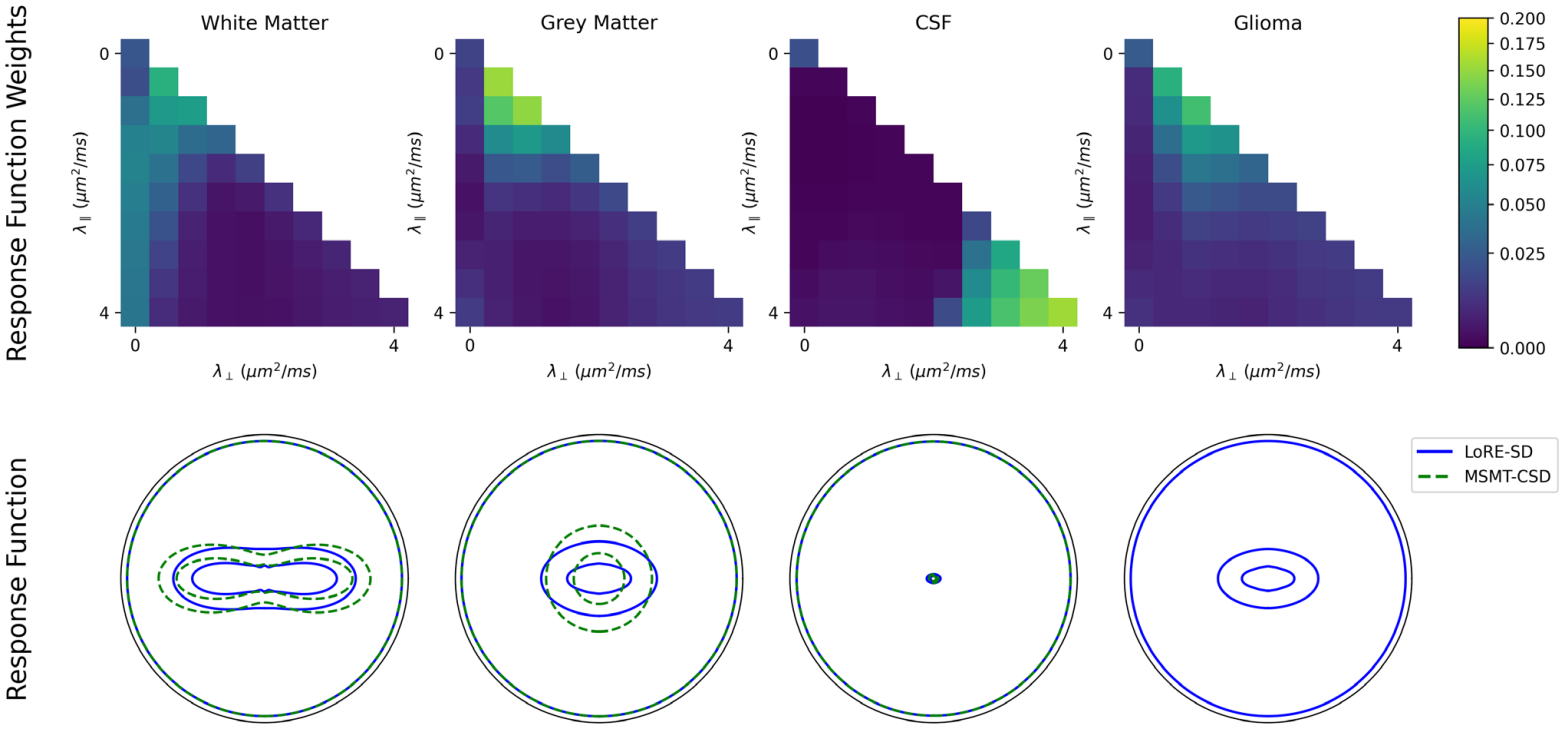
Average tissue response function representation (top row) and average response functions of LoRE-SD using a T1w tissue segmentation. White matter weights focus on low radial diffusivities, grey matter, and CSF focus on isotropic diffusivities. Glioma has a large variance in weight distribution. In addition, the MSMT-CSD response functions are overlaid on the average response functions as a dashed green line. The white matter response function of LoRE-SD is slightly more isotropic. The GM response function in MSMT-CSD is assumed to be isotropic, whereas LoRE-SD shows a noticeable amount of anisotropy. A glioma response function was not provided for MSMT-CSD due to high heterogeneity in glioma tissue and the lack of heuristics for response function estimation.

In addition to the contrast maps in Figure 5, the average tissue response functions are approximated and compared with MSMT-CSD in Figure 6. A T1w image is segmented into white matter, grey matter, and CSF using FSL FAST (Y. Zhang et al., 2001) and mapped into diffusion space using a linear registration. The lesion is manually delineated and set as a separate tissue type. To mitigate partial voluming effects and small registration misalignments, a binary erosion step was applied to the tissue segmentations. The final segmentations were used to calculate the average response functions of LoRE-SD. It is apparent that MSMT-CSD estimates a slightly more anisotropic white matter response function. This is a consequence of the response function estimation, where highly anisotropic voxels representative of single fibre bundles are selected. In contrast, the average response function of LoRE-SD includes voxels with crossing fibres, which exhibit greater isotropy. Additionally, the GM response function in MSMT-CSD is assumed to be isotropic, whereas LoRE-SD shows a noticeable amount of anisotropy.

### 4.4 Tractography

Accurate ODF estimation is a crucial component of tractography, which plays a prominent role in presurgical planning (Essayed et al., 2017; Fernandez-Miranda et al., 2012). For MSMT-CSD (Jeurissen et al., 2014), tractography uses the ODF magnitude to establish a stopping criterion that differentiates real from spurious fibre estimations (Tournier et al., 2010). Specifically, if the ODF amplitude along a tract decreases below a predefined threshold during generation, the tract is prematurely terminated. Since LoRE-SD employs ODF normalisation, an appropriate local scale factor must be applied to the ODFs to distinguish real from spurious fibres based on an ODF amplitude stopping criterion. As shown in Section 4.3, the novel representation introduced by LoRE-SD enables the generation of image contrasts, which provide a flexible way to scale the ODFs, and the specific contrast used for tractography may depend on the user’s needs.

**Fig. 7.**
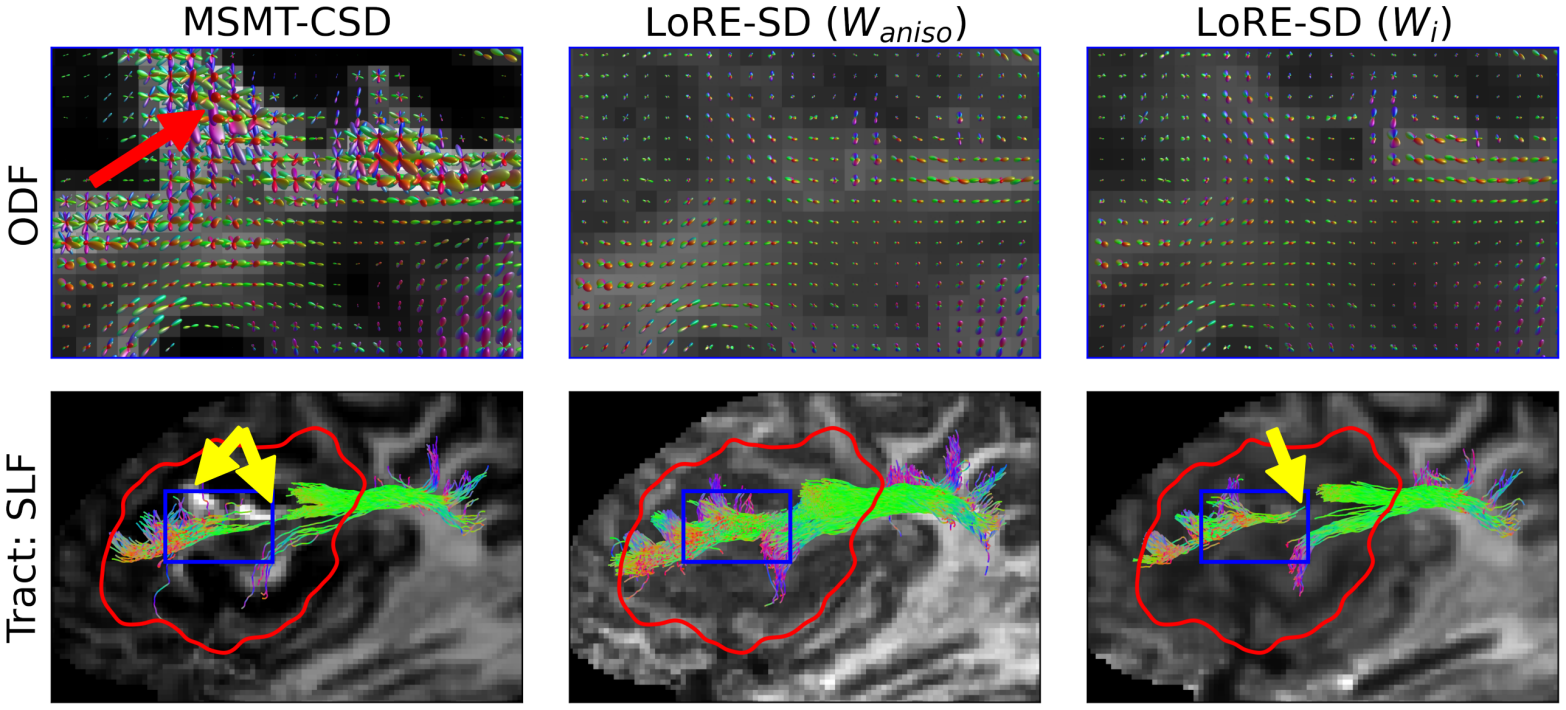
ODFS (top) and the reconstruction of the third division of the superior longitudinal fasciculus (bottom) in an oligodendroglioma dataset using MSMT-CSD and LoRE-SD. LoRE-SD ODFs are scaled by the response function anisotropy contrast (middle) and intra-axonal contrast (right). The contrasts are shown in the background. ODF estimations of MSMT-CSD and LoRE-SD align closely within the glioma (apart from the spurious ODFs by MSMT-CSD indicated with a red arrow), but the presence of isotropic compartments suppresses the ODF magnitude, which results in early track termination (yellow arrows). The response function anisotropy index is less strict towards higher radial diffusivities, which makes it more resilient against (near-)isotropic compartments and improves tractography.

Figure 7 shows the reconstruction of the right superior longitudinal fasciculus (SLF) in subject 2, a patient with oligodendroglioma. This reconstruction uses MSMT-CSD and LoRE-SD, with LoRE-SD ODFs modulated by either intra-axonal contrast ($W_{i}$) or response function anisotropy ($W_{aniso}$ ).

In areas where glioma infiltrates white matter, as shown in Figure 7, MSMT-CSD tends to overestimate the white matter volume fraction due to the high (isotropic) restriction to diffusion (red arrow). Consequently, the ODFs become excessively large and contain numerous spurious lobes, negatively impacting tractography by introducing spurious fibres in these regions.

Moreover, within glioma tissue, small ODFs of both MSMT-CSD and LoRE-SD when using intra-axonal modulation, lead to inadequate fibre tracking due to the stopping criterion (yellow arrows). In contrast, using the anisotropy scaling factor in LoRE-SD mitigates this issue, resulting in a clean SLF segmentation through the glioma region.

**Fig. 8.**
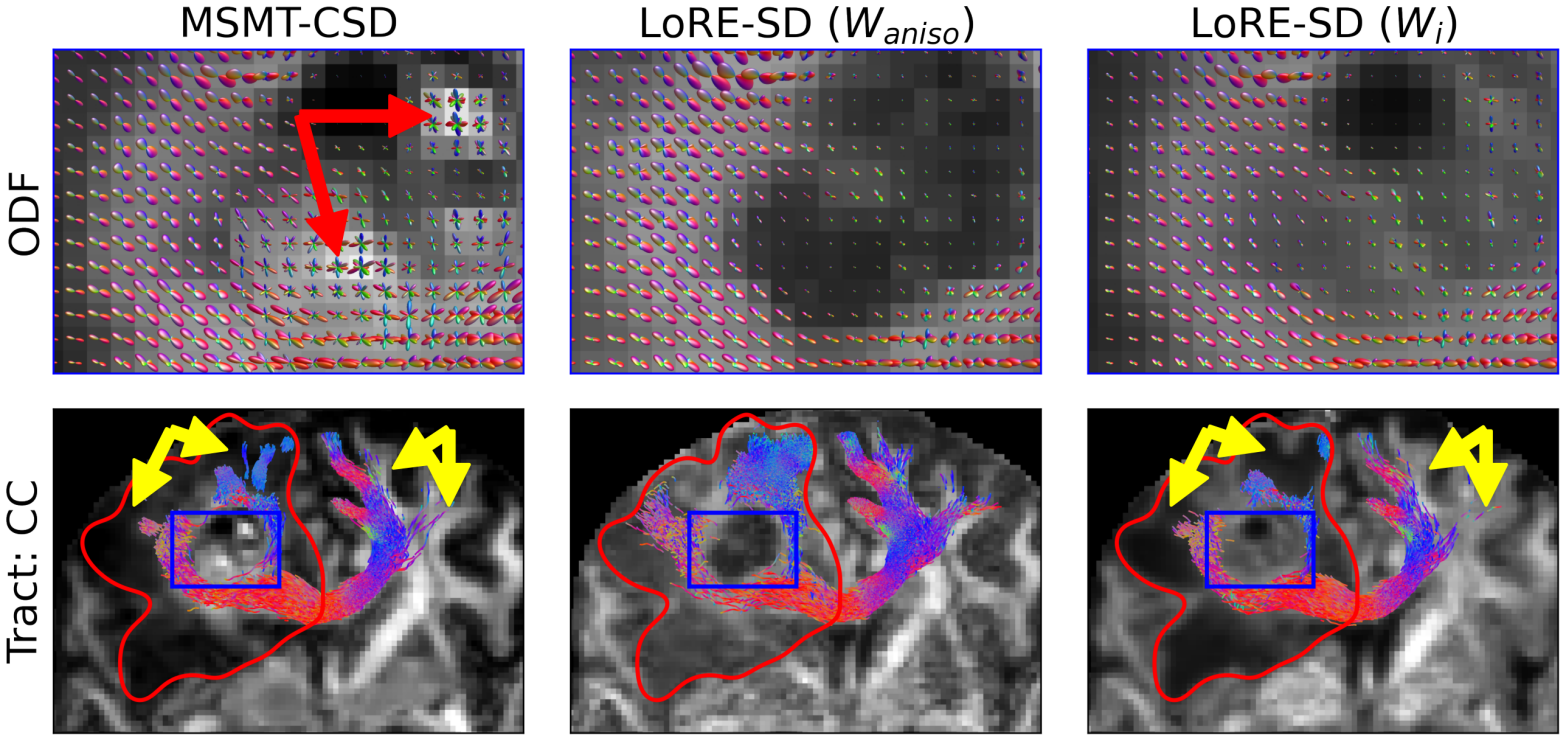
ODFS (top) and the reconstruction of the premotor cortex and supplementary motor cortex of the corpus callosum (bottom) in a frontal high-grade glioma dataset using MSMT-CSD and LoRE-SD. LoRE-SD ODFs (from the blue box in tractography) are scaled by the response function anisotropy contrast (middle) and intra-axonal contrast (right). MSMT-CSD produces spurious ODFs within the glioma (red arrows). Furthermore, MSMT-CSD and LoRE-SD with intra-axonal scaling ($W_{i}$) fail to track fibres to the cortex (yellow arrows) due to high ODF attenuation caused by oedema. The response function anisotropy ($W_{aniso}$ ) modulation does not suffer from this limitation.

Figure 8 depicts the reconstruction of the premotor cortex and supplementary motor cortex of the corpus callosum in subject 7 (frontal high-grade glioma). MSMT-CSD produces spurious ODFs within the glioma (red arrow). Furthermore, MSMT-CSD and LoRE-SD with intra-axonal scaling fail to track fibres to the cortex (yellow arrows) due to high ODF attenuation caused by oedema. Using the anisotropic scaling factor (middle column) resolves this.

**Fig. 9.**
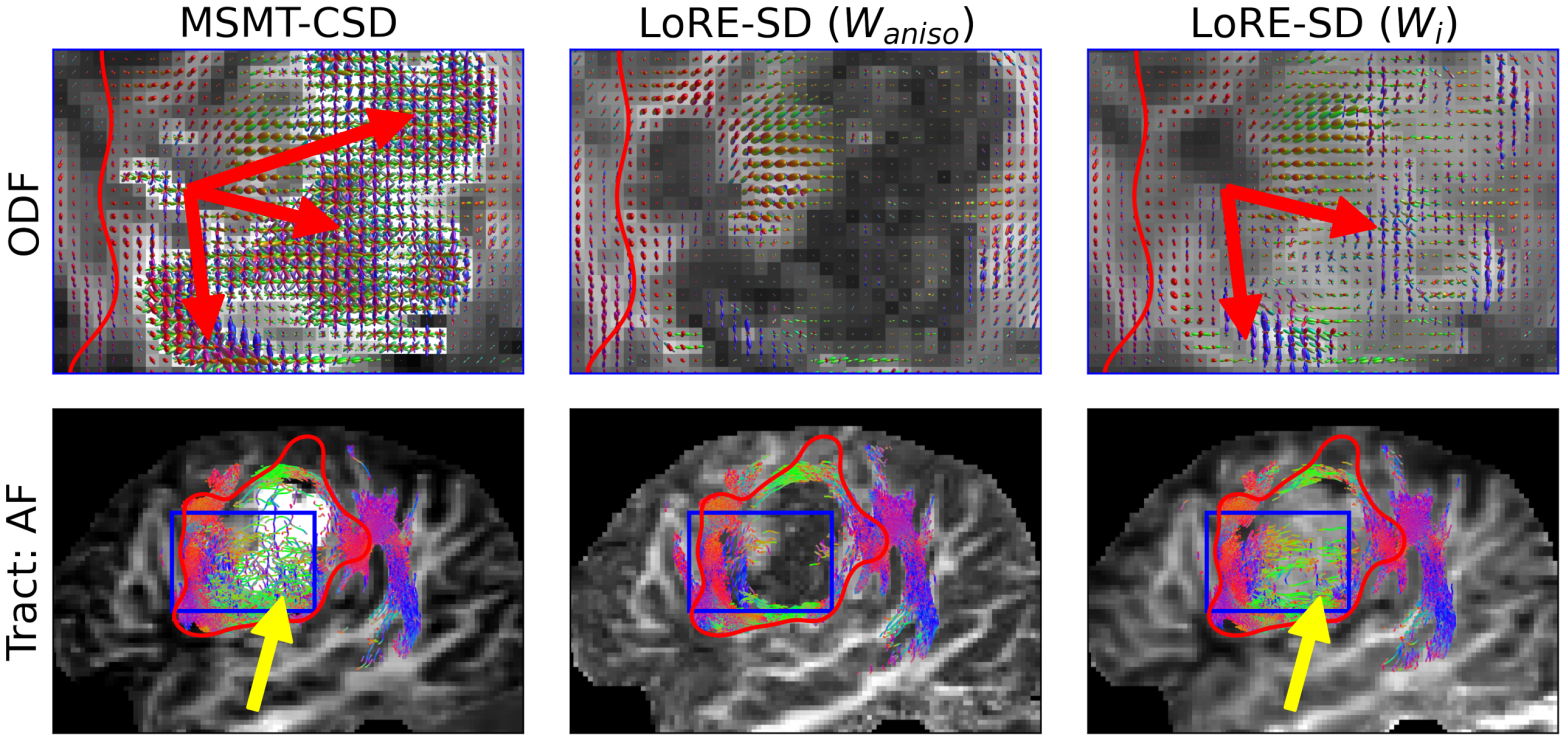
ODFS (top) and the reconstruction of the arcuate fasciculus (bottom) in a glioblastoma dataset using MSMT-CSD and LoRE-SD. LoRE-SD ODFs (from the blue box in tractography) are scaled by the response function anisotropy contrast (middle) and intra-axonal contrast (right). Water movement is highly restricted within the glioma, yet fairly isotropic. MSMT-CSD captures this restricted movement using the white matter response function, which explains the high WM volume fractions within the lesion and results in spurious tracking within the glioblastoma. Using LoRE-SD-derived ODFs and modulations results in less ($W_{i}$, right) or minimal spurious fibre tracking ($W_{aniso}$ , middle). The red arrows highlight spurious ODFs, while the yellow arrows highlight spurious fibre tracking.

Figure 9 depicts the reconstruction of the arcuate fasciculus in subject 8. MSMT-CSD utilises the white matter response function to model the highly restrictive compartments within the glioblastoma, leading to increased white matter volume fractions, spurious ODFs (red arrows), and the tracking of spurious fibres within the tumour (yellow arrows). Using intra-axonal contrast modulation (right) results in more accurate ODFs, but an overestimation of ODF magnitude, which also results in some spurious tracking. Response function anisotropy ODF modulation does not produce spurious tracks within the glioma.

A rating scale from 1 to 5 was used to validate the tractography results using visual assessment. An expert neuroradiologist responsible for neurosurgical planning visually scored all tractography outcomes. Results are presented in Table 2. Overall, using LoRE-SD with response function anisotropy modulation was the preferred approach in 7 out of 10 subjects. Subject 5 showed no clear differences between methods and MSMT-CSD was the preferred approach in subjects 1 and 6.

**Table 2.** Visual assessment of tractography in glioma patients using a rating scale between 1 and 5.

Subject	MSMT-CSD	LoRE-SD ($W_{aniso}$ )	LoRE-SD ($W_{i}$)
0	3	4	3
1	4	3	3
2	3	4	3
3	3	4	2
4	3	4	3
5	4	4	4
6	4	3	4
7	3	4	2
8	2	4	3
9	3	4	3
Mean	3.2	3.8	3.0

## 5 Discussion

This paper has introduced a novel method that enables data-driven estimation of a local response function within the spherical convolution paradigm, referred to as LoRE-SD. This approach offers a more generic dMRI signal representation compared with existing microstructure models due to its data-driven nature, allowing for accurate fitting of the data, even in the presence of pathology. Additionally, we use the response function representation to define novel contrasts related to the tissue microstructure, which may provide guidance for tractography.

### 5.1 Implications of a data-driven, local response function representation

A data-driven, local response function, presented here as a mixture of Gaussian basis functions, aims to accurately represent the data at hand by minimising assumptions on tissue composition or homogeneity. This makes data-driven approaches applicable in a broad range of datasets, independent of tissue types or tissue heterogeneity. As such, it is different from biophysical models, which impose strict assumptions on the microstructural compartments and their parameters (Assaf & Pasternak, 2008; Coelho et al., 2019, 2022; Jelescu et al., 2016; Jespersen et al., 2007; Kaden, Kruggel, et al., 2016; Lampinen et al., 2020; Novikov et al., 2019; Palombo et al., 2020; H. Zhang et al., 2012).

The functional form of biophysical models makes interpretation of the model parameters rather straightforward (keeping in mind potential biases due to model assumptions). However, a data-driven representation should implicitly capture the same information in the response function. The representation enables very flexible contrast generation, as shown in Figure 5, but, without further contrast matrix optimisation and validation, does not provide direct quantification of tissue compartments.

### 5.2 Disentangling the ODF and local response function

Evaluations using voxel-level and whole-brain simulations, as well as in vivo evaluations, demonstrate that LoRE-SD accurately models the diffusion signal. The performance improvement over MSMT-CSD is particularly compelling in pathological data. A priori defined tissue-average response functions introduce errors when modelling glioma as there is no clear average response function that captures the heterogenous behaviour of glioma. A data-driven, local estimation of the response function provides a more general solution, which is confirmed by AIC within glioma and oedema. Within healthy adult brain tissue, AIC shows that tissue-average response functions are sufficient and the additional model complexity of LoRE-SD is not justified. However, the versatility of the response function representation for contrast generation may also prove helpful in healthy tissue.

LoRE-SD disentangles the rotation-invariant microstructure from the fibre orientation distribution in each voxel, similar to data-driven approaches (Anderson, 2005; Jeurissen et al., 2014) and biophysical models (Behrens et al., 2003; Coelho et al., 2022; Kaden, Kruggel, et al., 2016; Novikov, Veraart, et al., 2018). LoRE-SD assumes that a single spherical convolution accurately describes the diffusion signal, which has been validated in vivo (Christiaens et al., 2020). This assumption is common in SD methods and embedded in most microstructure models and implies that multiple fibre bundles with different diffusion profiles within a voxel cannot be separated without hard constraints. To achieve a reliable disentanglement of ODF and response function, we introduce a regularisation term to promote more isotropic response functions, which has a similar effect as ODF sparsity regularisation (Daducci et al., 2014; Schultz & Groeschel, 2013), but has reduced computational complexity. In addition, the ODF is unit-normalised, such that the response function scales with the dMRI magnitude and the regularisation parameter λ is independent of this magnitude, providing a robust default value. Furthermore, the regularisation value of $\lambda =10^{-3}$ proved to be robust for a varying number of shells and gradient directions (results not shown).

The regularisation term assumes that any decrease in signal anisotropy stems from an increase in isotropic diffusion in microstructure, which is reflected by a more isotropic response function. Distinguishing perfectly isotropic fibre dispersion from a more isotropic fibre response is an inherently ambiguous problem in the spherical convolution paradigm, akin to the scaling ambiguity between the response function and the ODF. The chosen regularisation favours sparse ODFs and better resolves crossing fibres, which is argued to benefit tractography.

### 5.3 A generalised tissue representation for contrast generation and tractography

The novel response function representation, which is a weighted mixture model of Gaussian basis functions, enables LoRE-SD to construct scalar feature maps by highlighting basis functions of interest. Example contrasts are inspired by intra-axonal, extra-axonal, free water, and anisotropy maps. The contrasts shown in this work indicate LoRE-SD’s flexibility in generating a broad range of tissue contrasts. The maps are generated as the inner product between a contrast matrix W and the response function representation, which is a matrix of weights $f_{\lambda_{∥},\lambda_{⊥}}$. Thus any contrast matrix can be employed and future research may focus on contrast matrices that generate compartment-specific or pathology-specific contrasts. The representation may also be used as an input feature to learn a classification or segmentation of brain tissue or pathology.

The integration of a local response function with the Orientation Distribution Function (ODF) creates a flexible framework for tractography. Scalar contrasts derived from the response function representation provide various scaling factors for the ODFs and makes them compatible with ODF magnitude-based stopping criteria. By choosing specific feature maps, such as response function anisotropy or intra-axonal volume fraction, researchers can balance the trade-off between specificity and sensitivity in the tractography algorithm, potentially enhancing the accuracy of neural pathway mapping.

### 5.4 Limitations and considerations

While the results are encouraging, it is essential to acknowledge and address the inherent limitations and considerations associated with this work.

The representation of the response function assumes Gaussian diffusion within each compartment and does not allow inter-compartmental exchange. These are common assumptions in WM models, but may not hold in GM, where we may need to account for water exchange across cell membranes and the non-Gaussian diffusion along neuronal and glial processes (Jelescu et al., 2022). We argue that the inclusion of non-Gaussian diffusion components further increases LoRE-SD’s complexity and that modelling exchange and non-Gaussian diffusion would require a multi-dimensional data acquisition, for example, including multiple diffusion times, which should be the topic of future work.

The choice of grid size for the Gaussian basis functions plays an important role. In this work, these functions are defined on a square grid of equally spaced values of axial and radial diffusivity. However, it may be beneficial to use a non-uniform distribution with increased sampling density of the radial diffusivity on the low end. This approach could potentially provide a more precise representation of fibre density variations, thereby improving the subsequent analysis while preserving an acceptable runtime. In addition, there are minimal differences in signal reconstruction and ACC at grid sizes 5×5 and above, see Appendix A.2. While a 5×5 grid is more computationally efficient, it may not provide the required resolution for accurate contrast generation. To capture more complex contrasts beyond intra-axonal, extra-axonal, and free water, especially those tailored to specific pathologies, the enhanced resolution and precision of a 10×10 grid may be required. Furthermore, to allow potential noise-related biases that can sometimes lead to an overestimated ADC in free water, the upper limit has been set to $4\;\mu m^{2}\text{​}/\text{​}ms$ . However, the size and bounds of the discrete grid can easily be changed and adjusted by user preferences.

### 5.5 Future prospects

The novel and more general response function representation introduced by LoRE-SD provides new ways to represent the dMRI signal. In particular, LoRE-SD makes minimal assumptions on the microstructure which can be compelling in applications beyond the healthy human brain. We have shown this for glioma specifically, but argue that LoRE-SD may also prove beneficial in other neuropsychiatric diseases, in the developing human brain, in other organs, or in preclinical data.

The contrasts presented are not quantitatively compared with contrasts provided by biophysical models, as an accurate separation of tissue compartments requires multi-dimensional data. Future work may extend the response function representation to include non-linear tensor encoding (Lampinen et al., 2020; Novikov, Veraart, et al., 2018) and multiple echo time (Veraart et al., 2018). This may enable researchers to quantitatively validate LoRE-SD contrasts with microstructure models.

Different scaling factors of the ODF can significantly influence tractography outcomes, offering both benefits and challenges, as shown in Figures 7, 8, and 9. This strengthens the idea that there is no one-size-fits-all and that particular care has to be given to the interpretation of tractography (Daducci et al., 2016). However, with this in mind, the flexibility provided by LoRE-SD for ODF modulation provides researchers with the means to more thoroughly investigate its effect on tractography. Furthermore, the strategic application of ODF modulation may potentially refine tractography results but also support more detailed and customised analyses in both research and clinical settings.

## 6 Conclusion

In this study, we introduced LoRE-SD (Local Response function Estimation in Spherical Deconvolution), a novel approach that combines insights from microstructure modelling and the constrained spherical deconvolution paradigm. Our method provides a local, data-driven estimation of the response function, minimising assumptions about tissue microstructure. Specifically, by adopting a more generic model for the dMRI signal, we achieve improved accuracy in representing glioma tissue. Importantly, the applications of LoRE-SD may extend beyond glioma to other pathologies and organs.

Furthermore, the local response function representation serves as a valuable resource for developing novel contrasts and enhancing fibre tracking in the presence of pathology. Our framework details the methodology for creating these contrasts from the representation, including examples accentuating intra-axonal, extra-axonal, and free water spaces, and response function anisotropy. Compared with existing approaches, this enables a more diverse set of contrasts and thus more flexible tractography.

## Figures and Tables

**Appendix Fig. A1.**
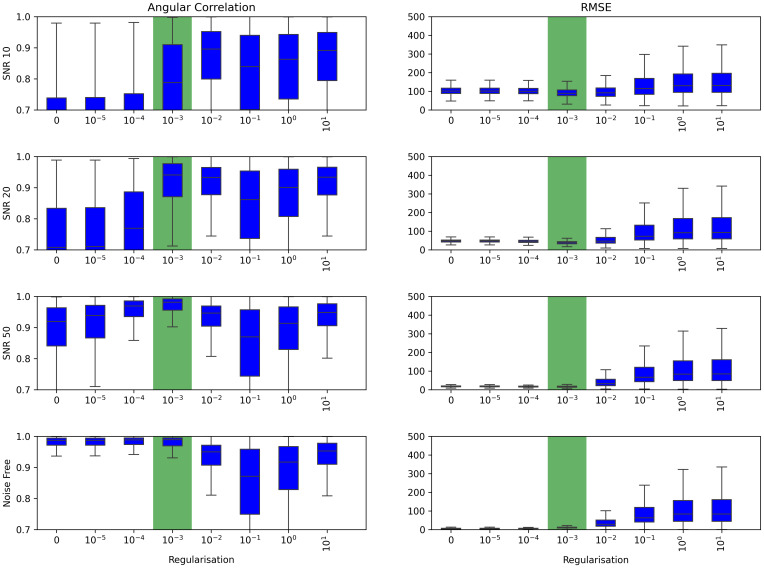
ACC and RMSE with respect to the regularisation parameter λ in full-brain simulations at different SNRs. The reference value (MSMT-CSD) and ground truth value are shown in different colours. The green band at $\lambda =10^{-3}$ was determined to provide an optimal trade-off between ODF accuracy and data fidelity. At $\lambda =10^{-3}$ there is a clear peak in ODF accuracy, while the RMSE only shows a marginal increase.

**Appendix Fig. A2.**
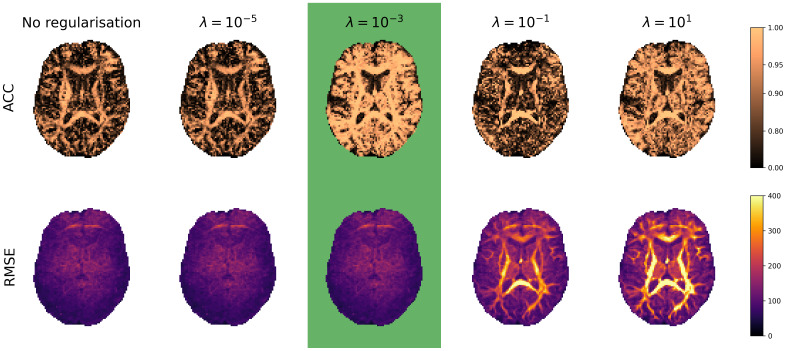
ACC and RMSE with respect to the regularisation parameter λ in in vivo data. The optimal trade-off between ODF accuracy and RMSE was determined at $\lambda =10^{-3}$ which is indicated as a green band. Using stronger regularisation results in more isotropic response functions, at which the ODF can no longer be retrieved accurately.

**Appendix Fig. A3.**
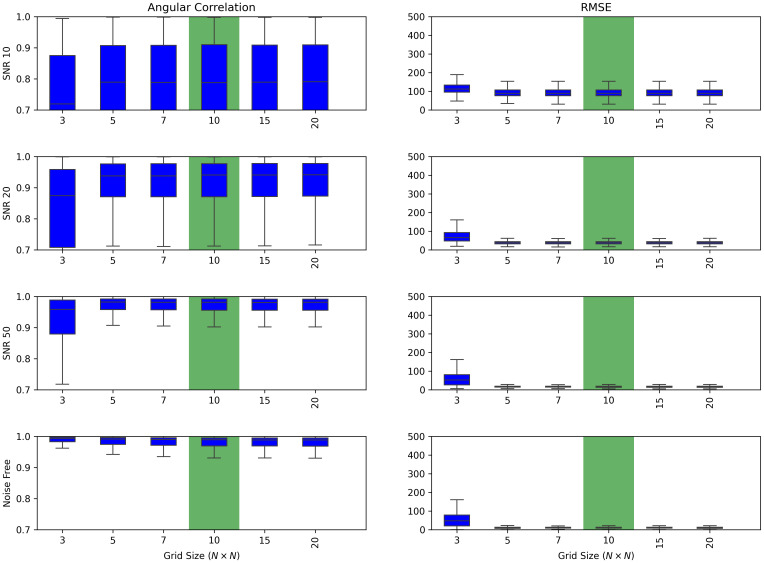
ACC and RMSE with respect to the grid size in full-brain simulations at different SNRs. The reference value (MSMT-CSD) and ground truth value are shown in different colours. The green band at a grid size of n=10 was determined to provide an optimal trade-off between ODF accuracy and data fidelity. While the differences between a grid size of n=5 and above seem neglectable, we argue that the grid size should not be too coarse to provide accurate contrast maps.

## Data Availability

The data that support the findings of this study are available on request from the corresponding author. The data are not publicly available due to privacy or ethical restrictions. The code is publicly available at: https://github.com/SiebeLeysen/LoRE-SD.git This code includes notebooks to replicate the voxel-level simulations and scripts to use LoRE-SD on in vivo datasets.

## A. Appendix

### A.1. Optimal regularisation parameter

The regularisation parameter λ determines the trade-off between the data fidelity term and the response function’s degree of anisotropy. A more isotropic—or smooth—response function should result in less spurious ODF peaks. However, there is no direct relationship between response function smoothness (or ODF sparsity) and ODF accuracy. If the response function is too isotropic, the spherical deconvolution can no longer accurately unravel crossing fibres and only a single fibre tract, if any, will be recovered. Furthermore, if the response function is perfectly isotropic, the ODF will not reflect any fibre tracts at all. This discrepancy between ODF sparsity and ODF accuracy makes that the l-curve method (Hansen, 2001) is not applicable to determine the regularisation parameter.

To determine the optimal value of λ, we re-consider the full-brain simulations for different noise levels. We balance ODF accuracy, quantified using the ACC between the estimated and ground truth ODF, and the RMSE of signal reconstruction. Figure A1 presents the ACC (in white matter) of LoRE-SD for varying regularisation strengths as well as the ACC distribution of MSMT-CSD. In addition, this figure presents the RMSE of LoRE-SD in function of λ, the RMSE of MSMT-CSD, and the ground truth RMSE.

The optimal value of $\lambda =10^{-3}$ is shown in green. ODF estimations are most accurate for this particular value, while the RMSE is in line with lower regularisation values, indicating that the regularisation does not affect the data fidelity. In addition, this figure confirms the hypothesis that a further increase in regularisation results in less accurate ODFs. At regularisation parameters larger than $\lambda =10^{-3}$ , the response function is (almost) isotropic and ACC values drop.

The optimal value of $\lambda =10^{-3}$ is also observed in in vivo data, see Figure A2. ACC values are visibly the highest in WM for $\lambda =10^{-3}$ , while the RMSE is in line with smaller regularisation values. At $\lambda =10^{-2}$ , we observe reduced ACC values in WM accompanied by an increase in RMSE in the deep WM. This observation persists at $\lambda =10^{-1}$ and above, where ODF estimations are no longer accurate and the RMSE in WM is significantly higher than in GM and CSF, indicating that the regularisation is too strong and the response function becomes isotropic, meaning that ODF can no longer be accurately estimated.

The regularisation value of $\lambda =10^{-3}$ proves to be robust against variations in the gradient table, both the number of shells and the number of gradient directions (data not shown).

### A.2. Optimal grid size

The response function is modelled as a linear combination of Gaussian basis function sampled on a grid of axial and radial diffusivities. The ranges of the axial and radial diffusivity have been fixed: $0\leq \lambda_{∥},\lambda_{⊥}\leq 4\;\mu m^{2}\text{​}/\text{​}ms$ . However, the resolution of the grid, defined by the number of equally spaced basis functions within the range, must be set appropriately. A grid that is too sparse will result in an inaccurate fit to the data and inaccurate parameter estimation. A grid that is too fine will result in a substantial increase in degrees of freedom, which leads to inefficient use of computational resources, and may be prone to overfitting.

Given a square grid of size n×n , the number of coefficients equals the nth triangular number: $\frac{n\left(n+1\right)}{2}$. This shows that the number of coefficients increases quadratically with respect to the grid resolution n. Figure A3 again makes use of the full-brain simulation; λ is fixed to $\lambda =10^{-3}$ . Using only three equally spaced values is clearly too coarse, while there is little difference between grid sizes of 5 and above. The difference is especially compelling at increasing SNR. The green band indicates the optimal value, namely 10 . Given this figure, there seems to be very little difference between a grid size of 5 or 10 , however, we argue that a coarse grid size of 5, resulting in only 15 weights, negatively impacts the interpretation of contrast maps derived from the representation.
